# A Quantitative Comparison of Calibration Methods for RGB-D Sensors Using Different Technologies

**DOI:** 10.3390/s17020243

**Published:** 2017-01-27

**Authors:** Víctor Villena-Martínez, Andrés Fuster-Guilló, Jorge Azorín-López, Marcelo Saval-Calvo, Jeronimo Mora-Pascual, Jose Garcia-Rodriguez, Alberto Garcia-Garcia

**Affiliations:** Department of Computer Technology, University of Alicante, Carretera San Vicente s/n, San Vicente del Raspeig 03690, Alicante, Spain; fuster@dtic.ua.es (A.F.-G.); jazorin@dtic.ua.es (J.A.-L.); msaval@dtic.ua.es (M.S.-C.); jeronimo@dtic.ua.es (J.M.-P.); jgarcia@dtic.ua.es (J.G.-R.); agarcia@dtic.ua.es (A.G.-G.)

**Keywords:** camera calibration, RGB-D, accuracy, Kinect, depth sensor

## Abstract

RGB-D (*Red Green Blue and Depth*) sensors are devices that can provide color and depth information from a scene at the same time. Recently, they have been widely used in many solutions due to their commercial growth from the entertainment market to many diverse areas (e.g., robotics, CAD, etc.). In the research community, these devices have had good uptake due to their acceptable level of accuracy for many applications and their low cost, but in some cases, they work at the limit of their sensitivity, near to the minimum feature size that can be perceived. For this reason, calibration processes are critical in order to increase their accuracy and enable them to meet the requirements of such kinds of applications. To the best of our knowledge, there is not a comparative study of calibration algorithms evaluating its results in multiple RGB-D sensors. Specifically, in this paper, a comparison of the three most used calibration methods have been applied to three different RGB-D sensors based on structured light and time-of-flight. The comparison of methods has been carried out by a set of experiments to evaluate the accuracy of depth measurements. Additionally, an object reconstruction application has been used as example of an application for which the sensor works at the limit of its sensitivity. The obtained results of reconstruction have been evaluated through visual inspection and quantitative measurements.

## 1. Introduction

Broadly, in a 3D vision system, three main stages can be identified: acquisition, data processing and analysis. All of these stages are constrained by the application requirements. In the analysis stage, useful measures of the data are obtained depending on the requirements of the final application. The data processing stage modifies the data in order to align the acquired views. In the first stage, the data is acquired by the sensor, therefore it is crucial because its quality affects later stages.

In order to meet the requirements, the acquisition stage is constrained by three parameters [1]: the scene, the subject of interest and the camera. The light, shadows or the point of view are some factors of the scene that affect the captured data. For example, most sensors that project a pattern to determine the depth of the scene are limited to working indoors, because they would not be able to identify the pattern under intense sunlight. The subject of interest also affects the acquisition, for example, it could include specular surfaces causing the reflection of the pattern. Finally, the acquisition is affected by the camera, which is conditioned by its sensitivity, calibration and technology.

Focused on the sensor, the acquisition of 3D data could be performed with different types of devices, broadly classified into two groups:

- *Contact devices.* They need a direct contact with the subject of interest to provide 3D information.
- *Contactless devices.* They are able to provide 3D information from the distance.

This paper is focused on the second group, specifically on optical sensors because they are faster, more flexible and can provide complementary information about the scene’s colour. Comprehensive reviews of these sensors for 3D measurement have been presented in several papers [2,3,4,5]. Moreover, different taxonomies have been proposed to classify this kind of sensors as the interesting unifying framework proposed by Davis et al. [6]. However, this group has been widely classified into passive and active methods [4,7,8]:

- *Passive methods* measure the scene radiance as a function of the object surface and environment characteristics using (usually) non-controlled ambient light external to the imaging system. Hence, only visible features of the scene are measured, providing high accuracy for well-defined features, such as targets and edges. However, unmarked surfaces are hard to measure [9]. In this category, techniques such as shape-from-X (e.g., shading, defocus, silhouettes, etc.), structure-from-motion and stereo are included. *Stereo vision* has received significant attention over the past decade in order to provide more accurate results and obtain them faster [10]. Usually, the methods use two or more calibrated RGB cameras to get the depth image by computing the disparity information from the images that conform to the system [11]. Stereoscopic cameras have been used for many purposes, including 3D reconstruction [12]. This technology can provide both colour and depth information, but it is required to be calibrated every time its location is changed, making its portability more difficult. Besides, they need the presence of texture to obtain the 3D information. In some devices, the distance between both cameras could be changed to fit the working range of the system.
- *Active methods* use their own light source in the imaging system for the active illumination of the scene [13]. The sensor is usually focused on known features from this light source. Then, the illumination and the features are designed to be easily measured in most environments. Since they have difficulties with varying surface finish or sharp discontinuities such as edges [9], compared with the passive approach, active visual sensing techniques are in general more accurate and reliable [14]. Active sensors could be classified into two broad categories [15]: triangulation and time delay. The former rely on the triangulation principle using the light system, the scene and the sensor. The main differences between the methods include the nature of the controlled illumination (laser or incoherent light) and its geometry (beam, sheet, or projected pattern). Laser triangulators, structured light and moiré methods are examples that fall into this level. Time delay systems measure the time between emission and detection of light reflected by the scene (Time-of-flight, ToF) or the phase difference between two waves (Interferometry). Focusing on the ToF, pulsed-light and continuous wave modulation are the technologies available nowadays. Pulsed-light sensors directly measure the round-trip time of a light pulse. In order to obtain a range map, they use either rotating mirrors (LIDAR - Light Detection and Ranging o Laser Imaging Detection and Ranging) or a light diffuser (Flash LIDAR). LIDAR cameras usually operate outdoors and their range can be up to a few kilometers. Continuous wave sensors measure the phase difference between the emitted and received signals and usually operate indoors. Thier ambiguity-free range is usually fixed from 30 cm to 7 m [16,17]. A extensive comparison of ToF technologies can be found in [18].

**Figure 1:**
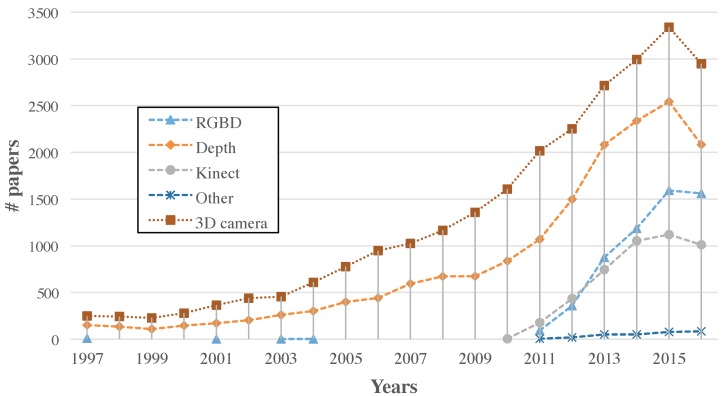
Number of publications calculated from Scopus containing 3D imaging, depth cameras, RGB-D cameras, Microsoft Kinect sensors and other devices (including Asus Xtion, Primesense and Intel RealSense).

Depending on the application requirements, a specific imaging device is selected according its characteristics. A comprehensive review including advantages and disadvantages for different applications of the most important techniques and sensors for the optical 3D measurement of a scene was presented by Sansoni et al. [5]. One of the conclusive remarks of the study was that most of the equipment available was significantly expensive, being an obstacle to a much wider distribution of 3D systems. However, they observed a trend towards a decrease in costs due to the increased competition of manufacturers and the technology evolution decreasing costs. One year later, popularized by Microsoft releasing the first generation of Kinect in November 2010 and focused on the entertainment market, consumer RGB-D (*Red Green Blue and Depth*) sensors emerged, which underlines this fact. As we can see in Figure 1, the introduction of Kinect sensor boosted the number of publications related to depth cameras and 3D imaging systems in general. Although the RGB-D topic was named in a few papers before 2011, it is in that year when the technological term was adopted, after the first Kinect version appeared. Moreover, the number of papers citing the Kinect camera has represented on average about 77% of the research publications on RGB-D sensors. These sensors combine one of the previous techniques (*Structured Light, Time-of-Flight...*) with an RGB camera to provide the colour and depth images of the scene using a common CMOS sensor for the color image and a different infrared technology to acquire the depth information.

RGB-D devices are mainly characterized by their low cost [19,20,21] but also they provide portability, high framerate, multidimensional perception with good accuracy for a wide range of applications. Hence, the use of these sensors has grown and been generalized from home entertainment systems to areas like robotics, medical informatics, etc. [22,23]. However, in some applications a calibration process could be needed in order to increase their accuracy, because they work in the limit of its sensitivity and some characteristics of the subject of interest might not be perceptible.

Khoshelham and Elberink [20] carried out a study into the accuracy of the Microsoft Kinect sensor. Some works propose algorithms and applications using RGB-D sensors. Han et al. [24] carried out a review focused on the Microsoft Kinect, but a more general review could be found in [25]. In [26], the authors performed a comparative of registration methods for RGB-D sensors. Weiss et al. [27] used a Microsoft Kinect to obtain a 3D model of a subject from multiple views around the body, avoiding the use of expensive devices. In [28], the authors were able to obtain a 3D model of a foot from multiple images around it using a PrimeSnese Carmine 1.09 and augmented reality markers. Jedvert [29] also used a Microsoft Kinect to obtain a 3D model of the head with hight quality textures. The work of Paier [30] aims to obtain a 3D model of a face for subject identification in security systems. In [31], the authors demonstrated that default parameters used by a Microsoft Kinect are not good enough for many applications.

To increase the accuracy of the data provided by these sensors, some works perform a calibration process. In [32], the authors propose an algorithm to calibrate the intrinsic parameters of both cameras, providing the necessary information to convert the disparity to meters. Zhang and Zhang [33] extend this work looking for correspondences between colour and depth images of a calibration pattern. Burrus [34] performed the calibration of a Kinect sensor based on the OpenCV calibration to obtain the intrinsic and extrinsic parameters. In [31], the authors noticed residual errors in depth images after calibration, estimating a fixed error for each pixel and calculating a correction pattern. Later, Daniel Herrera et al. [35] proposed a distortion correction on disparity images, taking into account that the magnitude of this error decreases as the distance from the subject increases. Subsequently, Raposo et al. [36] improved that proposal using fewer images for the input of the calibration process. Recently, Staranowicz et al. [37] proposed a method to estimate the parameters of a depth camera from images of a spheric object, using the Hough transform and performing a non linear minimization to obtain the results.

From the study of the state-of-the-art practice, it can be demonstrated that RGB-D sensors are useful for many applications, but their sensitivity is not suitable for problems in which higher accuracy of the data is required. To the best of our knowledge, there exist works carrying out a comparative of calibration methods for RGB-D sensors. For example, Staranowicz and Mariottini [38] made a comparison of three calibration methods [32,33,37] and, recently, Xiang et al. [39] did the same for others [34,35,40]. Meanwhile, Lachat et al. [13] made a comparison of the provided raw data, a calibration method and photogrammetry. Also, it is important to highlight the work carried out by Staranowicz et al. [41] in which they propose a comparative of different calibration methods using different known objects: checkerboard and spheres. However, all of them performed the evaluation of the methods and sensors using only a single consumer RGB-D sensor, the Microsoft Kinect v1 or v2. Analysing the technologies used by RGB-D sensors and the available calibration algorithms to improve their accuracy are very important topics that could be addressed simultaneously (i.e., quantifying the result of each algorithm in different technologies). In order to do so, in this paper, a comparative study of calibration algorithms applied to RGB-D sensors is presented in order to analyse the accuracy limits of this sensors.

The rest of the paper is structured as follows: in Section 2 the common technologies used by these sensors are explained and so are the three most common calibration methods. Section 3 shows the calibration results for each sensor and the developed experiments to test the accuracy for each one. An example of object reconstruction is used in order to evaluate the accuracy of the results in an application wherein the sensor works at the limit of its sensitivity. Finally, in Section 4 the conclusions of this work are shown.

## 2. Materials and Methods

In this section, a description of the RGB-D cameras and the calibration methods used in the quantitative comparison are presented.

### 2.1. RGB-D Cameras

**Figure 2:**
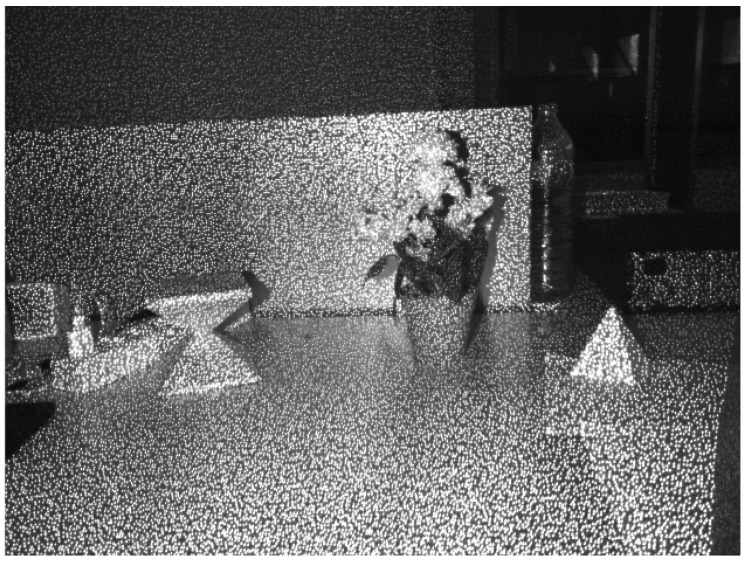
Projected pattern by Microsoft Kinect.

**Figure 3:**
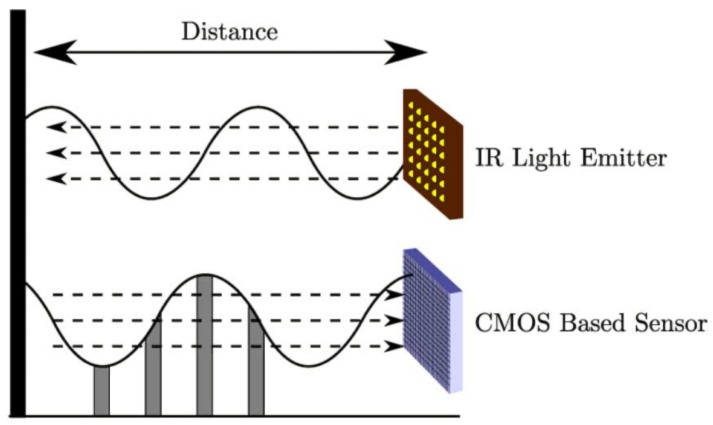
Time-of-Flight distance measurement [16].

Popularized by Microsoft, releasing the first generation of Kinect in November 2010 focused on the entertainment market, RGB-D sensors have been improved by different companies. Nowadays, many sensors could be found to provide depth. Table 1 shows the technical specifications of different low cost RGB-D cameras (price is less than 200 euros). Generally, consumer RGB-D cameras are mainly based on Structured light and ToF:

- Structured Light (*SL*) based sensors are composed of a near-infrared emitter and an infrared (IR) camera. The infrared emitter projects a known pattern over the scene, simultaneously the IR camera gets the pattern and computes the disparity between the known and the observed pattern [42,43,44]. Usually, the infrared is chosen as the bandwidth of the projected pattern to avoid interfering with visible light in the scene. Nevertheless, a drawback of this technology is the impossibility of working in places where the illumination hinders the perception of the pattern [45]. More information about this technology can be found in [20]. For example, consumer RGB-D as Microsoft Kinect, Asux Xtion Pro or PrimeSense Carmine use structured light by projecting a speckle pattern over the scene (see Figure 2).
- Time-of-Flight (*ToF*). As has previously been stated, ToF sensors obtain the distance to a subject of interest by measuring the time between the emission of a signal and its reflection from the subject. Consumer cameras that use this technology are based on a continuous wave sensor combined with a calibrated and internally synchronized RGB camera. A near-infrared emitter emits incoherent light, which is a modulated signal with a frequency *ω*. This light incises in the scene, producing a reflected signal with a phase shift ϕ with respect to the emitted signal (see Figure 3). Hence, the distance is given by the Equation (1), where *c* is the speed of light [46]. Microsoft Kinect V2 is the best representative example of this kind of cameras, achieving one of the best image resolutions among ToF cameras commercially available and an excellent compromise between depth accuracy and phase-wrapping ambiguity [18].
  (1) $$d=\frac{c\phi }{4\pi \omega }\;$$

In this study, Microsoft Kinect, Primesense Carmine 1.09 and Microsoft Kinect v2 have been selected. Structured light and ToF technologies for RGB-D cameras are represented by these sensors. Specifically, Kinect sensors have been selected because they are the most used and popular RGB-D cameras in the research community nowadays. As it was suggested by Figure 1, they represent the most papers dealing with this kind of sensors. The Microsoft Kinect V2 sensor has significant differences compared to its previous version. It is based on ToF technology with better resolution of 1920 × 1080 for the colour camera, but keeps the operation range of the depth camera. In case of the Primesense Carmine 1.09, although it is based on the same principle as Microsoft Kinect v1 and ASUS Xtion as popular cameras (in fact, it is the same Primesense patent [47] and uses the same PS1080 processor developed by the Primesense company), the operation range is different. The Carmine 1.09 sensor is a short range sensor, so its depth camera can operate between 0.35 m and 1.4 m, approximately, while the Microsoft Kinect works between 0.5 m and 4.5 m. The goal to include this sensor is to analyse the use of a specific short range device compared to the standard measuring range of the popular kinect devices for applications that require a short range, such as 3D reconstruction, for example.

### 2.2. Camera Calibration Parameters

Camera calibration is a necessary step in 3D computer vision in order to extract metric information from images [48]. It enables the determination of the camera geometric and optical characteristics and/or the relative position and orientation of the camera frame with respect to a world coordinate system [49]. One of the criteria that has to accomplish the calibration procedure is to be accurate because it is necessary to infer accurate 3D information from images. In other words, the calibration goal is to provide the measured data as close as possible to the real value. The more accurate the calibration model is, the more accurate the data that is provided by the camera system.

The calibration parameters of the cameras could be divided into two groups. One of them refers to those parameters which are specific to the lens geometry (intrinsic parameters), and those that are related to the relative position and orientation of the camera frame (extrinsic parameters). We refer to Hartley and Zisserman [50] for an extensive work of multi-view geometry in computer vision where the parameters are studied. For the sake of completeness, we briefly introduce the most relevant ones for this work below.

#### 2.2.1. Intrinsic Parameters

**Figure 4:**
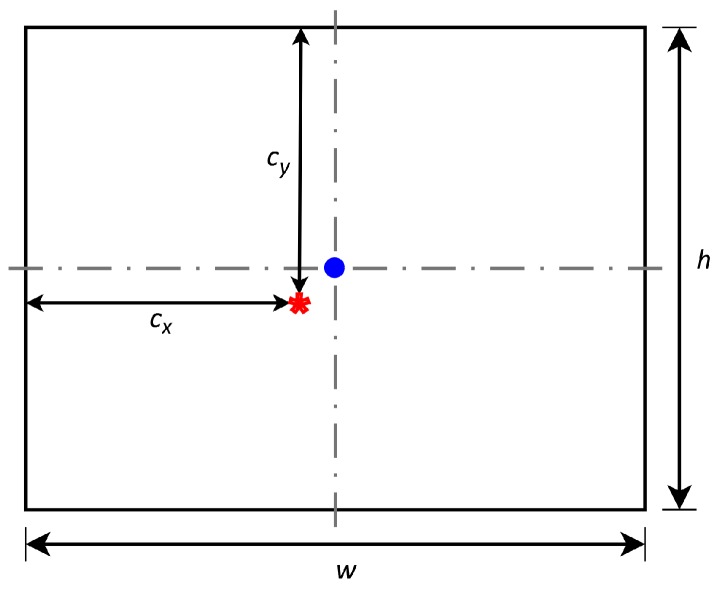
Visual representation of the principal point.

**Figure 5:**
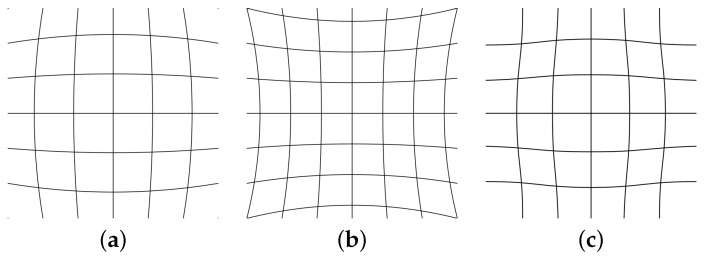
Different models of optical distortions. (**a**) Barrel [51]; (**b**) Pincushion [52]; (**c**) Moustache [53].

Intrinsic parameters refer to the internal camera geometric and optical characteristics: focal length, distance between the optical centre of the lens and the photosensitive sensor; the principal point, represents the displacement of the optical axis, producing a displacement of the projection centre in the image (see Figure 4); and the distortion coefficients, which is the optical distortion model of a camera (see Figure 5). The latter refers to the variation of a straight projection due to the aberration of the lens. It is zero in the principal point, and increases with the distance.

The 3D point cloud is then calculated using the focal length and the principal point parameters, along with the depth image. The distortion is corrected to accurately provide the 3D data reducing the lens shape defects. For further details about the 3D point set estimation and optical parameter modeling, refer to Appendix A.

**Figure 6:**
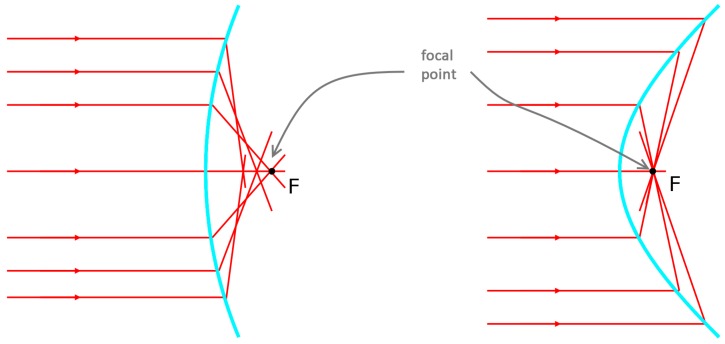
Difference between spherical lens (**left**) and parabolical lens (**right**).

The distortion coefficients represent the optical distortion model of a camera. The two most common are the radial and the tangential ones. This is produced by the imperfect parabolic shape of the lenses, which are more spherical, producing the misalignment of the rays and resulting in a distorted image (see Figure 6).

#### 2.2.2. Extrinsic Parameters

Extrinsic parameters refer to the relative position and orientation of the camera frame with respect to a world coordinate system. Specifically, in a multiple camera system, such as the stereo cameras or the RGB-D sensors studied here, multiple images are obtained from different coordinate systems. In this case, the extrinsic parameters describe the geometric relationship between the cameras that might be needed. Schulze [54] presented several calibration methods and discussed the accuracy to calibrate extrinsic parameters for aligning range sensors and colour cameras. For stereo matching, or RGB and Depth matching, it is necessary to align those images to a common coordinate system. This matching is carried out using the extrinsic parameters, which define the rotations and translations, the *baseline* which is the distance between the sensors, and the orientation of each camera because they are not perfectly parallel each other. For further information about the extrinsic parameters modeling, refer to Appendix A.

### 2.3. Calibration Methods

There are several methods to calibrate 3D sensors, most of them can be applied to RGB-D cameras. A comprehensive overview of the current approaches adopted for camera calibration in close-range photogrammetry and computer vision could be found in [55]. According to Xiang et al. [39], these methods can be classified in supervised and unsupervised calibration. The first ones perform the process acquiring images of targets with a particular shape or size, while unsupervised methods use the environment. This paper is focused in the first group, supervised calibration, due to the performance of unsupervised methods usually being lower. Besides, the methods can be distinguished between classical and those that are focused on 3D cameras of different technologies, ToF, SL, stereoscopic, etc. Usually, 3D cameras have two independent lenses and employ a technology to compute the depth. Classical methods calibrate cameras with one lens. Tt could be applied to calibrate each lens independently obtaining the intrinsic parameters, but they do not provide the extrinsic ones. Moreover, some calibration methods for RGB-D sensors are able to calibrate the parameters tat are employed to compute the depth information, which is specific to this technology.

A classification of calibration methods can be found in [41] including supervised and non-supervised calibration methods. This paper is focused on the most common supervised methods to calibrate 3D sensors (see Table 2). Usually, they use a set of images of a pattern composed of squares, known as *chessboard* or *checkerboard*. The corners of the chessboard are easily detected by a corner detector algorithm, but other kinds of patterns could be used. The methods have been evaluated according to a set of characteristics: the year of publication; the number of citations obtained; if the method performs a joint calibration, which is the calibration of both cameras simultaneously; the input data required by the algorithm; the type of target employed in the images; if the target is known by the algorithm or not; the number of images required to calibrate the sensor and the availability of the code.

The results obtained by Xiang et al. [39] showed that the best results where obtained with the methods of Daniel Herrera et al. [35] and Burrus [34]. In this paper, these two methods have been selected for the comparative. Moreover, the method of Bouguet [56] has been included in the comparative, due to it having been widely used in the literature and, in contrast to the other methods, the calibration of the infrared camera has to be performed with the infrared images only.

#### 2.3.1. Bouguet Method

Bouguet [56] published a generic method for camera calibration based on the work of Zhang [64], Zhang [48] and Tsai [49]. The algorithm proposed by Zhang [64] only requires images of a planar pattern at different orientations. Later, this algorithm was adapted to work with 3D cameras [48], while Tsai [49] proposed a tow stage technique also for 3D camera calibration. The Bouguet method also includes an add-on to calibrate stereoscopic systems that allows us to calibrate both colour and depth cameras of an RGB-D sensor. Smisek et al. [31] used this method to calibrate a Microsoft Kinect, while Van Den Bergh and Van Gool [65] did the calibration of a ToF sensor with a colour camera coupled to it. The input to the calibration algorithm are the colour and infrared images obtained simultaneously.

**Figure 7:**
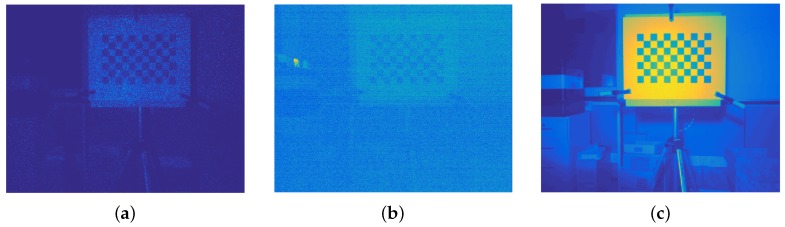
Infrared (IR) images of the chessboard. (**a**) Infrared image of the pattern; (**b**) Infrared image of the pattern without IR emitter; (**c**) Infrared image of the pattern without IR emitter and using a light bulb.

Due to the IR emitter, the obtained infrared images are very noisy (see Figure 7a) and the corners of the calibration pattern can not be detected properly. In the images obtained without the IR emitter (Figure 7b) the chessboard is not perceptible because the image is very dark. In order to get images that could be used in the calibration process, a light bulb focused to the chessboard is needed (see Figure 7c).

#### 2.3.2. Burrus Method

*RGB Demo* [34] is a set of tools and libraries to work with the data provided by a Microsoft Kinect sensor, but also could be used with devices supporting the same driver. One of the included tools is for calibrating this device using the calibration algorithm implemented in OpenCV based on Bouguet [56] and Hartley [66]. The calibration process is performed as if it were a stereoscopic system. Firstly, RGB, infrared and disparity images are obtained to calibrate the intrinsic parameters of both cameras individually. Then, a stereoscopic calibration process is done to get the extrinsic parameters.

#### 2.3.3. Herrera Method

The method proposed by Daniel Herrera et al. [35] to calibrate colour cameras and a depth camera simultaneously have been developed with the objective of being accurate, practical and applicable to multiple sensors. The algorithm implements the intrinsic error model of the Microsoft Kinect depth camera, but it could be replaced to work with similar devices.

The intrinsic error model allows us to correct the distortion of the depth camera in the disparity image, and is based on the constant error in depth measurements that appear in this kind of sensors. Besides, this error decreases when the distances to the sensor increase.

The result of the algorithm provides a spatial distortion pattern $D_{\sigma }$, which is a matrix of the same size of the depth image; the values $\alpha_{0}$, $\alpha_{1}$ that represent the decadence of the distortion effect with the distance, and the values $c_{1}$, $c_{0}$ to convert the disparity to meters. Then, the disparity for a given pixel (u,v) of the depth image can be corrected with Equation (2).

(2) $$d_{k}=d+D_{\sigma }\left(u,v\right)\cdot exp\left(\alpha_{0}-\alpha_{1}\dot{d}\right)$$

Where *d* is the disparity in the pixel (u,v) and $d_{k}$ is the corrected value of the disparity in that pixel. Once the disparity has been corrected, the distance in meters $Z_{d}$ for that pixel could be computed by Equation (3).

(3) $$Z_{d}=\frac{1}{c_{1}\cdot d_{k}+c_{0}}$$

## 3. Experimentation

**Figure 8:**
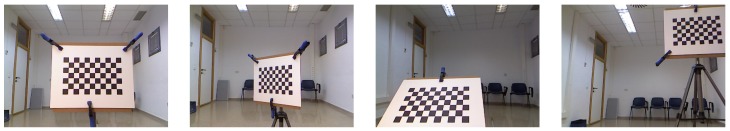
Some images of the chessboard used in the calibration process of Microsoft Kinect.

In order to comparatively analyse the performance of the Bouguet, Burrus and Herrera calibration methods, three different sensors have been used as was stated in Section 2: Microsoft Kinect, Primesense Carmine 1.09 and Microsoft Kinect V2. For each sensor, different images of a chessboard pattern varying its position and orientation from the camera have been acquired (see Figure 8). This pattern is composed of 7 × 11 squares of 0.034 m of size. A subset of 60 images for each camera have been selected, which have been used for the calibration methods.

### 3.1. Calibration Results

The results obtained with each calibration method for Microsoft Kinect and Primesense Carmine 1.09 are shown in Table 3 and Table 4, respectively. The results for Microsoft Kinect V2 are in Table 5. Note that the values for the principal point ($c_{x}$, cy) for the RGB camera obtained with the Burrus method for this sensor are not correct, it should be located near the center of the image with resolution 1920 ×1080, but the obtained coordinates are (345.85,251.59) due to the assumption of a fixed standard resolution. Additionally, the Microsoft Kinect V2 could not be calibrated using the method of Herrera because this camera can not provide the disparity images used by the method.

### 3.2. Experimental Results

We have carried out three experiments to evaluate the results of each calibration method. The plane fitting test was used by Khoshelham and Elberink [20] to evaluate the error in the distance, while with the measurements of the height and the markers, the accuracy of each method in combination with each camera is evaluated.

#### 3.2.1. Plane Fitting Test

**Figure 9:**
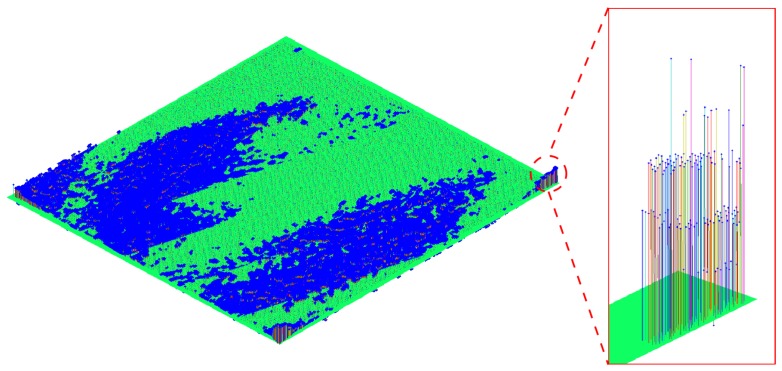
Plane fitting test, visual procedure. (Blue) 3D points of a wall. (Green) plane computed with RANSAC that best fit with the acquired points. The augmented part shows the point to plane orthogonal distances used to carry out this test.

We have obtained different images of a wall at various distances (0.7 m, 0.8 m, 0.9 m, 1 m, 1.1 m, 1.2 m and 1.3 m) with each sensor, applying the corrections and computing the point cloud with the parameters provided by each calibration method. Also, images without any correction have been used to compare the accuracy obtained with the default parameters, which are unknown. Due to the difficultly of placing the sensor perfectly parallel to a wall, a square of 100 × 100 pixels from the center of the image has been extracted (blue points in Figure 9) computing the best plane that fits those points (green plane in Figure 9) using RANSAC [67] (*Random Sample Consensus*). Then, the outliers have been removed and the point-to-plane orthogonal distances with the remaining points have been computed. The error has been computed as the distance *d* from a point $P=(x_{0},y_{0},z_{0})$ to a plane π≡Ax+By+Cz+D=0. This distance corresponds to the perpendicular line from the point to the plane and its given by Equation (4).

(4) $$\begin{array}{c} d\left(P,\pi \right)=\frac{\left|Ax_{0}+By_{0}+Cz_{0}+D\right|}{\sqrt{A^{2}+B^{2}+C^{2}}} \end{array}$$

**Figure 10:**
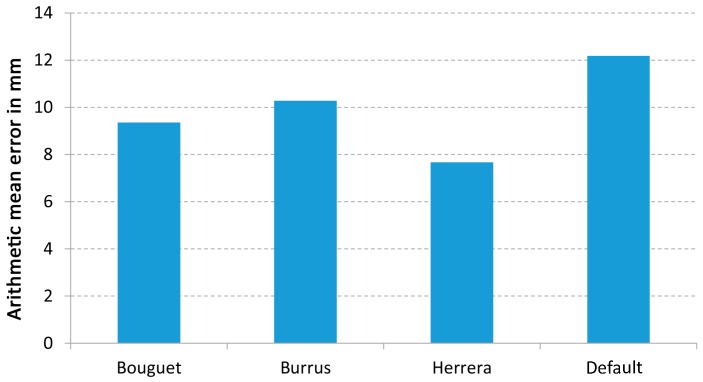
Plane fitting test error for each calibration method for all cameras.

Figure 10 shows the arithmetic mean error of each calibration method. As it can been seen, all methods improve the results obtained with the default parameters which gives an error of 12.18 mm. In particular, the method of Herrera provided the smallest error (7.67 mm), while the error for the Bouguet and Burrus methods were very close each other, 9.36 mm and 10.28 mm, respectively.

**Figure 11:**
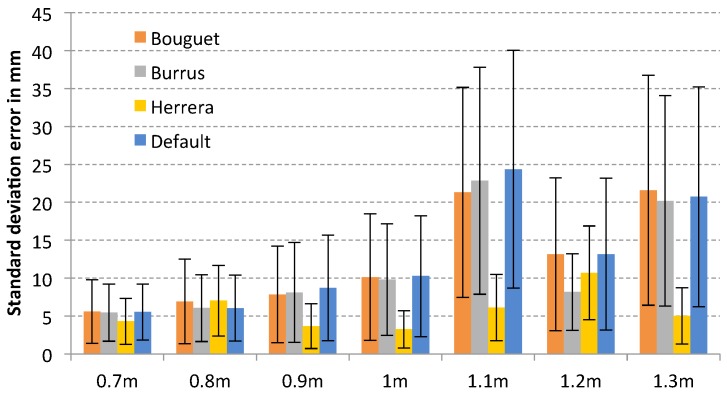
Plane fitting test error of each calibration method for Microsoft Kinect.

**Figure 12:**
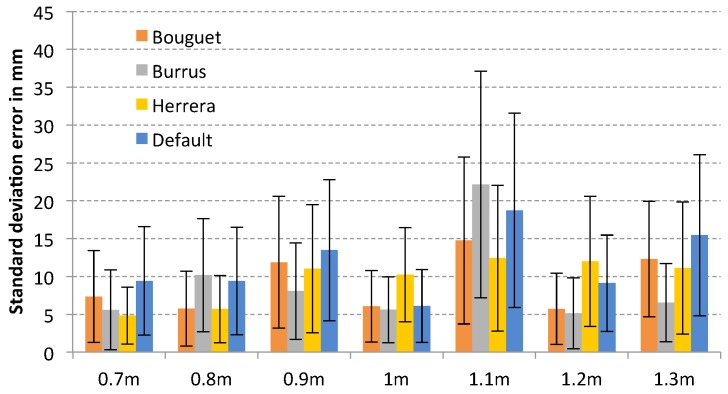
Plane fitting test error of each calibration method for Primesense Carmine 1.09.

**Figure 13:**
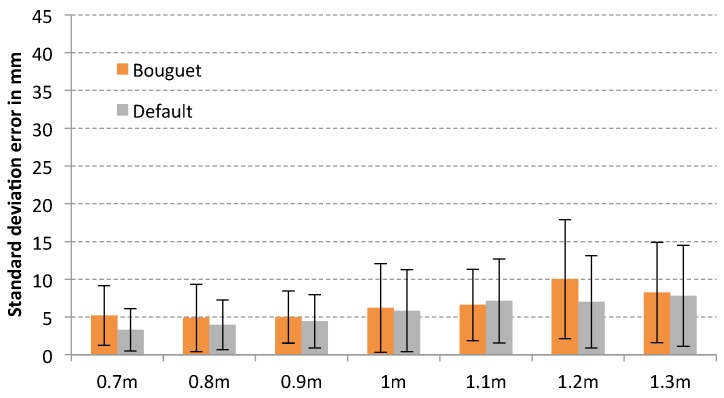
Plane fitting test error of each calibration method for Microsoft Kinect V2.

Analysing the standard deviation error for each sensor, it is possible to observe that the method of Herrera provides the best result in general terms for Microsoft Kinect (see Figure 11), with an std error of 5.73 mm, and Primesense Carmine 1.09 (see Figure 12), with an std error of 9.61 mm due to the distortion correction for the depth camera that provides this method. However, in the case of Primesense Carmine 1.09, the lowest average error is obtained with the Burrus method (9.044 mm). In case of Microsoft Kinect V2 (see Figure 13) there is not much difference between the default results and the calibrated ones, but in most cases the smallest error has been obtained with the default parameters. This is because the error in the depth is evaluated in this test, but only the calibration of the internal parameters of the infrared camera is performed. The calibration of the depth computation in a ToF sensor is complex due to it being difficult to calculate the frequency of the modulated signal and the phase shift of the reflected one. For this reason, there is not much difference between the calibrated and the raw results. Nevertheless, the lowest error for the Bouguet method has been obtained with this sensor (5.20 mm) which is based on ToF.

#### 3.2.2. Measurement Error

**Figure 14:**
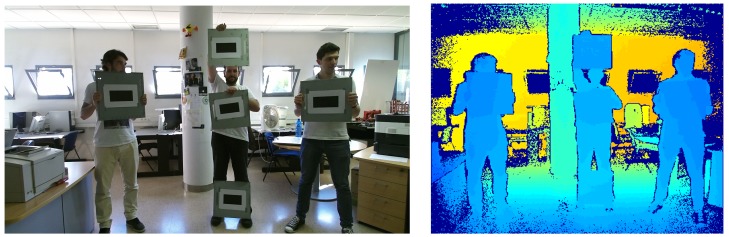
Color (**left**) and depth (**right**) images of the markers distributed in the image.

The accuracy of the measurements of planar targets of size 10 cm × 20 cm have been compared. The targets have been acquired at two different distances from the camera, 1.5 m and 2 m, distributed among the image space (see Figure 14). Then, the obtained images have been corrected with the parameters provided by the calibration methods, and the height and width of the markers have been measured, analysing the error with the real size. Then, the arithmetic mean for each sensor and method has been calculated.

**Figure 15:**
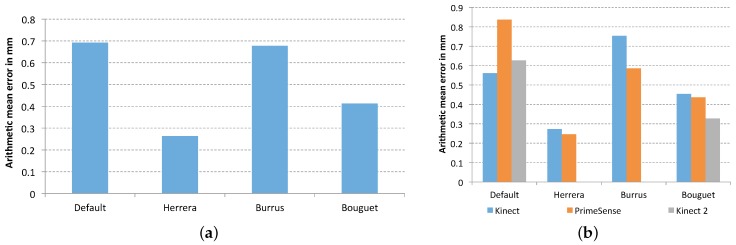
Accuracy of the measurements. (**a**) Error of each method; (**b**) Error of each sensor group by method.

Figure 15a shows the arithmetic mean error group by method obtained in the corrected data in comparison with that obtained with the default parameters. The smallest error is provided by the data corrected with the parameters of the method of Herrera (with a mean error of 0.26 cm), while the default parameters provide the higher error (0.69 cm). The difference between the Bouguet and Burrus method is bigger than in the previous experiment, with values of 0.41 cm and 0.67, respectively.

Looking at this arithmetic mean error according to various sensors in Figure 15b, the best result is obtained with the Primesense Carmine 1.09 calibrated with the method of Herrera. However, the results of Microsoft Kinect with the same method are very close. It is important to highlight the results obtained for each camera with the calibration method of Bouguet, in which the sensor based on ToF gets better results than those based on structured light.

#### 3.2.3. Object Registration

**Figure 16:**
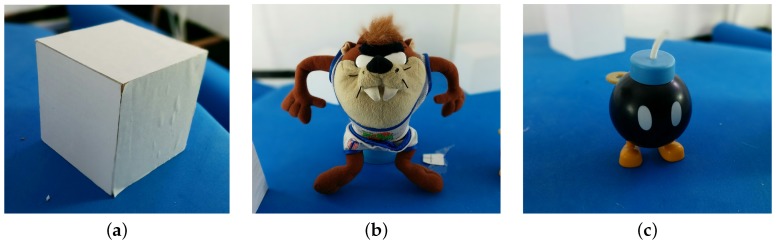
Registered objects. (**a**) Object 1 (Cube); (**b**) Object 2 (Taz); (**c**) Object 3 (Bob-omb).

**Figure 17:**
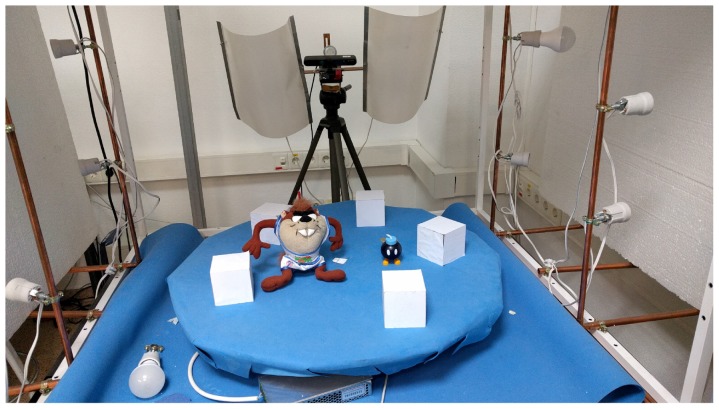
Controlled environment.

Additionally, since one of the most common applications for RGB-D sensor is 3D reconstruction, it has been used as example to compare the accuracy of each calibration method. This application is an example in which the sensor works at the limit of its sensitivity.In order to do that, the *μ*-MAR method [68] has been used, which performs a 3D reconstruction of an object from different views based on 3D markers (Figure 16a) to properly compute the transformation to align the views. Concretely, *μ*-MAR models the markers to reduce the effects of noise and register the scene with these models. This application is an example in which the sensor works at the limit of its sensitivity. The data have been acquired in a controlled environment (showed in Figure 17) including a set of 79 images for each object shown in Figure 16. The objects studied here are an 8 cm side cube (Figure 16a), that is one of the markers; Figure 16b is a 20-cm high Taz toy; a 5-cm Bom-omb toy shown in Figure 16c. The reason for using a marker as an object to analyze is because its registration quality is direct applied to the objects’ registration result, since the *μ*-MAR registration is based on the markers. The data from the acquisition has been corrected using the parameters from different methods, and then registered.

In order to evaluate the registration, visual inspection and a quantitative analysis have been performed. Regarding the visual inspection, we are going to pay attention to some details of the shapes to compare calibration methods. Concretely, the easiest shape is the cube. A section of the registered cube is presented to easily appreciate the quality of registration. Moreover, for the other objects, the arms of the Taz and the eyes of the Bob-omb are the regions that will be used to perceive the registration accuracy.

**Figure 18:**
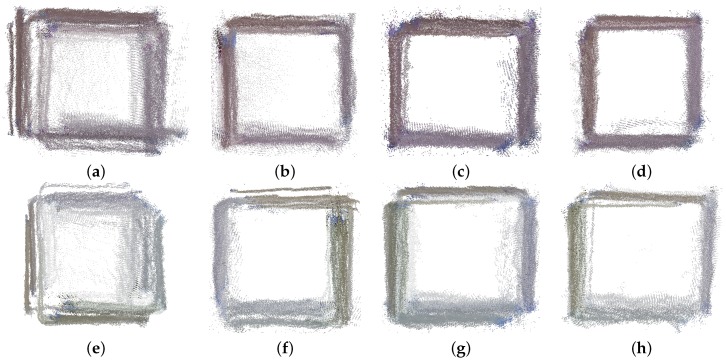
Section of the cube acquired with Kinect v1 in the first row and the Primesense in the second row. The section shows the cube seen from the top. (**a**,**e**) Default; (**b**,**f**) Burrus; (**c**,**g**) Bouguet; (**d**,**h**) Herrera.

The first experimentation presented is the cube object for the Microsoft Kinect v1. The registration results are presented in the first row of Figure 18. The different views are well aligned when they are perfectly overlapped. On the other hand, if there is an error in the registration, it is possible to see how a single view is displaced from the rest. The default calibration (Figure 18a is clearly the worst registered result since many views are wrongly registered. Bouguet, shown in Figure 18c, has some error, like the left side of the cube, where a view is not accurately registered. Burrus achieves good results but has some views on the top and left side, which are not adequately registered (a slim gap can be seen between views). Herrera achieves the best result providing the most compact and accurate registration presented in Figure 18d. The second row of Figure 18 shows the results for the data acquired using the Primesense. In this case, despite the result being better than in the Kinect v1, the default calibration (Figure 18e) is the worst in terms of registration result. For the rest of the calibration methods, the registration result has some errors (areas where cube sides are misaligned) with the Herrera being the best in general terms since the average shape is more square-like.

After the study of the cube, which shows in a simple view the main accuracy of the registration with data from different calibrations, the experimentation with the second and third objects (Figure 16c,d) are studied.

**Figure 19:**
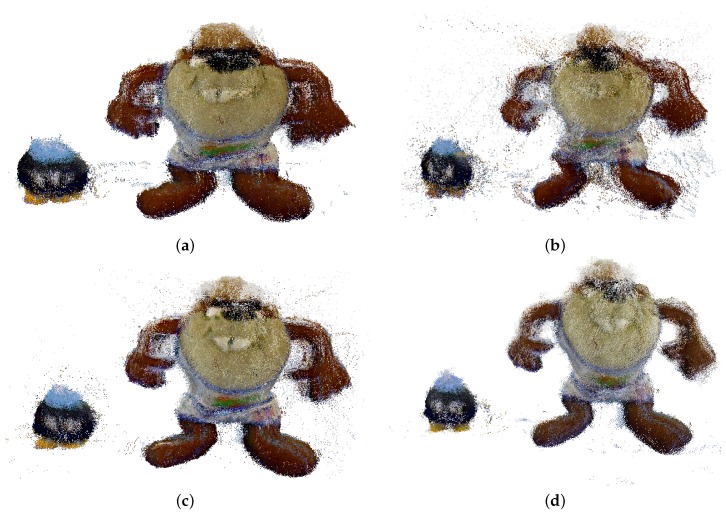
Frontal view of the reconstruction obtained with Primesense Carmine 1.09. (**a**) Default; (**b**) Burrus; (**c**) Bouguet; (**d**) Herrera.

The registration results using the Primesense Carmine 1.09 are presented in Figure 19. The data from Herrera (Figure 19d) achieves the best registration, confirming the previous experimentation. The Taz arms in this method are more compact and the Bob-omb eyes are better defined. Bouguet method Figure 19c) provides adequate data, but slightly worse (Bob-omb eyes and the spherical shape is distorted). Observing the original data, registration is clearly the worst in the four results, as is clear by looking at the poorly registered arms of the Taz.

**Figure 20:**
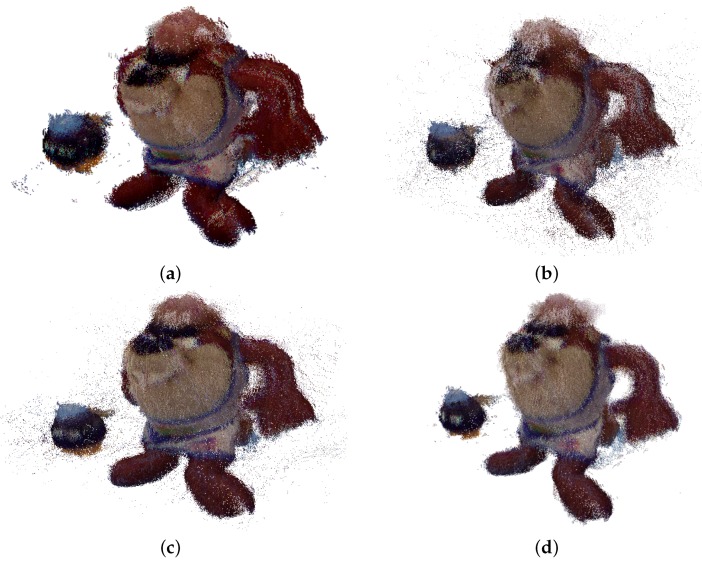
Perspective view of the registration obtained with Microsoft Kinect v1. (**a**) Default; (**b**) Burrus; (**c**) Bouguet; (**d**) Herrera.

**Figure 21:**
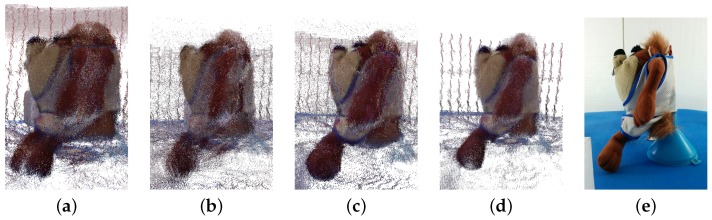
Side view of the reconstruction of the Object 1 obtained using different calibration methods with Microsoft Kinect v1. (**a**) Original; (**b**) Burrus; (**c**) Bouguet; (**d**) Herrera; (**e**) Real.

**Figure 22:**
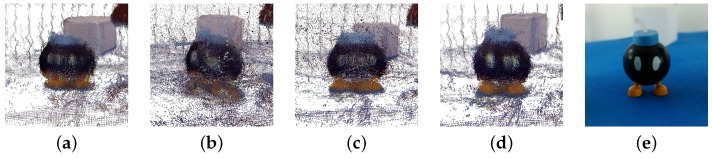
Frontal view of the reconstruction of the Object 2 obtained using different calibration methods with Microsoft Kinect v1. (**a**) Original; (**b**) Burrus; (**c**) Bouguet; (**d**) Herrera; (**e**) Real.

Similar results are obtained with the Microsoft Kinect v1, showed in Figure 20. Figure 21 and Figure 22 show a side view of Taz and frontal of Bob-omb, for the registration results. As before, the arm registration could be used as a visual feature to visually evaluate the performance of Taz and the eyes of Bob-omb. Again, the data provided by Herrera (Figure 21d and Figure 22d) calibration achieves the best registration.

**Figure 23:**
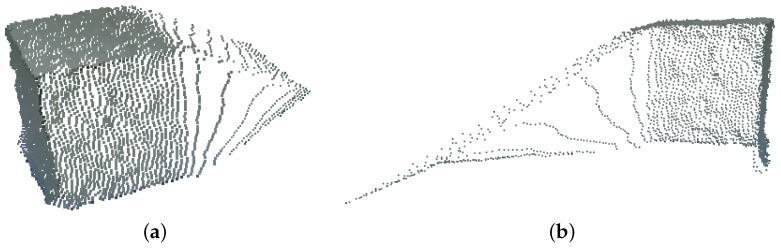
Noise distribution obtained with Kinect V2 in the acquisition of the cubes. **(a)** Perspective view; **(b)** Side view.

The Microsoft Kinect V2 RGB-D sensor has a particular noise distribution, presented in Figure 23. Due to this noise, the markers used by the μ−MAR registration method cannot be accurately modeled, and hence, the registration cannot be done. Since the cubes are formed by planar surfaces, the model is obtained as a set of planes with ninety degrees between each other, located by fitting the points of each face in the point cloud. However, as it is possible to see in Figure 23a,b that the faces of the cube are prolonged in a non planar direction (e.g., the top face leans downwards). This makes it impossible to fit a planar model in a face and hence the method algorithm does not provide satisfactory results.

**Figure 24:**
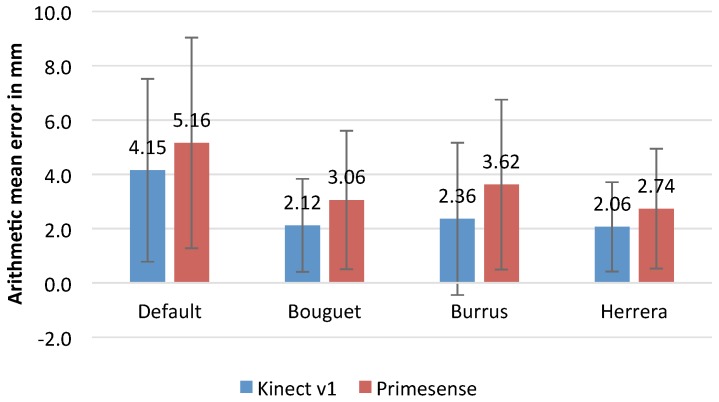
Registration error for different calibration methods in order to reconstruct a cube.

Finally, regarding the quantitative analysis, the study has been carried out using the cubes because the mathematical model of the shape to be reconstructed is known and can be used as the ground truth. Specifically, the euclidean distance from a point to the corresponding plane of the face of the cube has been used as a measure of the registration error. Figure 24 shows the average mean of distances for all points to the corresponding planes of the cube. The conclusions reached using visual inspection, related to the effect of calibration methods in the registration quality, are coherent with the measure of the error calculated by means of the mean point to plane distance. The highest registration error corresponds to the default calibration. Bouguet and Burrus have similar error levels. Finally, Herrera method achieves the lowest error. This conclusions are valid for both Kinect v1 and Primesense devices.

## 4. Conclusions

In this paper, three calibration algorithms have been compared by applying their results to three different RGB-D sensors. The obtained parameters for each camera haven been tested in different situations and the results have been analyzed. In addition, these parameters have been applied in 3D reconstruction of objects, which is an application for this kind of sensors where they work at the limit of their sensitivity. In the experiments, the results obtained through the calibration method of Herrera were the most accurate. Additionally, the best reconstruction was obtained with the data processed with this algorithm for Microsoft Kinect. In the reconstruction, the Microsoft Kinect sensor showed better results than the Primense Carmine 1.09, which is a short range camera. Besides, based on the quantitative experiments, it is possible to observe that from 1 meter to the structured light cameras, the error begins to increase. Moreover, the quantitative experiments showed the best results for Microsoft Kinect V2 applying the Bouguet method. However, it has not been possible to use this sensor for 3D reconstruction due to the flying pixels problem, which complicates the perception of the markers. Future works will include the use of other calibration methods, like the one proposed by Staranowicz et al. [37] and the denoising of the data acquired with Microsoft Kinect V2 so that it can be used with the μ−MAR method, and compare the results of both versions of Kinect.

## Figures and Tables

**Table 1:** Technical specifications of consumer RGB-D cameras. SL: Structured light, ToF: Time of Flight.

Sensor	Measuring Range (m)	Error	Field of View HxV (Degrees)	Resolution Colour/Depth	Depth Resolution (cm)	Technology	FPS
Kinect v1	0.8–3.5	<4 cm	57 × 43	640 × 480 640 × 480	1 @ 2 m	SL	15/30
Carmine 1.08	0.8–3.5	-	57.5 × 45	640 × 480 640 × 480	1.2 @ 2 m	SL	60
Carmine 1.09	0.35–1.4	-	57.5 × 45	640 × 480 640 × 480	0.1 @ 0.5 m	SL	60
Xtion Pro	0. 8–3. 5	-	58 × 45	1280 × 1024 640 × 480	1 @ 2 m	SL	30/60
Real Sense	0. 2–1. 2	1%	59 × 46	1920 × 1080 640 × 480	-	SL	30/60
Kinect v2	0. 5–4. 5	0.5%	70 × 60	1920 × 1080 512 × 424	2 @ 2 m	ToF	15/30
Senz3D	0. 2–1. 0	-	74 × 41. 6	1080 × 720 320 × 240	-	ToF	30

**Table 2:** Common supervised calibration methods (I = IR, D = Disparity, Z = Depth, C = Color).

Method	Year	Citations	Joint Calibration	Input Data	Type of Target	Known Target	Number of Images (Approx.)	Available Code
Daniel Herrera et al. [35]	2012	223	Y	D,C	Chessboard	Y	20	Y [57]
Zhang and Zhang [33]	2011	107	Y	Z,C	Chessboard	Y	12	Y [58]
[34]	2011	37	Y	I,Z,C	Chessboard	Y	30	Y [34]
Bouguet [56]	2004	2721	N	I,C	Chessboard	Y	20	Y [56]
Raposo et al. [36]	2013	30	Y	D,C	Chessboard	Y	10	Y [59]
Staranowicz et al. [37]	2014	13	Y	Z,C	Spheres	N	-	Y [60]
Tsai [49]	1987	7113	N	C	Flat surface with squares	Y	1–8	Y [58]
Fuchs and Hirzinger [46]	2008	150	N	Z	Chessboard + robotic arm	Y	50	N
Lichti [61]	2008	452	N	Z	Rectangular targets of different sizes	N	-	N
Jiejie Zhu et al. [62]	2008	251	N	Z	Chessboard	Y	-	N
Lindner and Kolb [63]	2007	76	N	Z	Chessboard	Y	68	N

**Table 3:** Calibration results for Microsoft Kinect.

	Burrus		Bouguet		Herrera	
	RGB Camera	IR Camera	RGB Camera	IR Camera	RGB Camera	IR Camera
$f_{x}$	523.24	595.99	523.16±1.40	588.18±1.58	522.55±0.25	586.80±0.45
$f_{y}$	521.68	592.44	521.32±1.35	586.00±1.52	520.24±0.25	577.70±0.59
$c_{x}$	328.65	314.43	330.14±1.04	315.83±1.21	329.76±0.33	318.92±0.35
$c_{y}$	257.03	227.05	257.01±1.14	245.20±1.25	257.59±0.37	231.46±0.37
$k_{1}$	0.0215	−0.1567	0.1475±0.00609	−0.0724±0.0052	0.1930±0.0024	0
$k_{2}$	−0.6927	0.6467	−0.2735±0.0116	0.1306±0.01	−0.5651±0.012	0
$k_{3}$	0.7170	−0.8859	0	0	0.4843±0.0176	0
$p_{1}$	−0.0007	0.0012	−0.0014±0.00082	0.0009±0.0007	−0.0006±−0.0004	0
$p_{2}$	−0.0005	0.0004	0	−0.0013±0.00071	−0.0003±0.0002	0
$c_{0}$	−	−	−	−	−	3.0946±0.0035
$c_{1}$	−	−	−	−	−	$-0.0028\pm 3.7100\times 10^{-6}$
$\alpha_{0}$	−	−	−	−	−	1.2521±0.0510
$\alpha_{1}$	−	−	−	−	−	$0.0022\pm 7.4073\times 10^{-5}$
*R*	$\left[\begin{array}{lll} 0.9995 & 0.0082 & -0.0052\\ -0.0081 & 0.9988 & 0.0125\\ 0.0053 & -0.0125 & 0.9999 \end{array}\right]$		$\left[\begin{array}{l} -0.0076\\ -0.0031\\ 0.0078 \end{array}\right]$ ± $\left[\begin{array}{c} 0.0012\\ 0.0016\\ 0.0004 \end{array}\right]$		$\left[\begin{array}{ccc} 1 & -0.0077 & -0.0047\\ 0.0077 & 0.9999 & -0.0084\\ 0.0048 & 0.0084 & 1 \end{array}\right]$ ± $\left[\begin{array}{c} 6.9160\times 10^{-4}\\ 5.9122\times 10^{-4}\\ 3.4263\times 10^{-4} \end{array}\right]$	
*T*	$\left[\begin{array}{l} -0.0255\\ 0.0026\\ 0.0068 \end{array}\right]$		$\left[\begin{array}{l} 0.0250\\ 0.0004\\ -0.0003 \end{array}\right]$ ± $\left[\begin{array}{r} 0.0001\\ 0.0001\\ 0.0004] \end{array}\right]$		$\left[\begin{array}{l} 0.0269\\ -0.0026\\ -0.0024 \end{array}\right]$ ± $\left[\begin{array}{c} 3.9870\times 10^{-4}\\ 4.5291\times 10^{-4}\\ 6.1674\times 10^{-4} \end{array}\right]$	

**Table 4:** Calibration results for Primesense Carmine 1.09.

	Burrus		Bouguet		Herrera	
	RGB Camera	IR Camera	RGB Camera	IR Camera	RGB Camera	IR Camera
$f_{x}$	540.84	580.04	540.58±0.64	575.46±0.68	541.67±0.16	574.98±0.23
$f_{y}$	539.48	576.45	538.95±0.62	573.98±0.65	539.48±0.16	570.58±0.31
$c_{x}$	318.38	307	318.62±0.99	318.79±1.05	316.87±0.27	323.97±0.23
$c_{y}$	237.82	232.75	238.32±0.86	245.13±0.90	235.48±0.24	227.71±0.2
$k_{1}$	0.0512	−0.0687	0.0232±0.0023	−0.0401±0.0029	0.0578±0.0015	0
$k_{2}$	−0.2236	0.2196	−0.0939±0.0059	0.0304±0.0061	−0.2610±0.0069	0
$k_{3}$	0.1785	−0.4167	0	0	0.2430±0.0098	0
$p_{1}$	0.0010	−0.0007	0.0012±0.00045	0.00011±0.00044	0.0003±0.0001	0
$p_{2}$	−0.0009	−0.004	−0.00064±0.00055	−0.00014±0.00054	−0.0017±0.0001	0
$c_{0}$	−	−	−	−	−	4.0054±0.0021
$c_{1}$	−	−	−	−	−	$-0.0029\pm 1.68\times 10^{-6}$
$\alpha_{0}$	−	−	−	−	−	1.6229±0.0304
$\alpha_{1}$	−	−	−	−	−	$0.0021\pm 4.06\times 10^{-5}$
*R*	$\left[\begin{array}{lll} 0.9999 & 0.0049 & 0.0089\\ -0.005 & 9.9992 & -0.005\\ -0.0089 & -0.0112 & 0.9989 \end{array}\right]$		$\left[\begin{array}{l} -0.00214\\ 0.00201\\ 0.00429 \end{array}\right]$ ± $\left[\begin{array}{c} 0.00089\\ 0.00121\\ 0.0001 \end{array}\right]$		$\left[\begin{array}{ccc} 1 & -0.0040 & 0.0086\\ 0.0042 & 0.9998 & -0.0169\\ -0.0086 & 0.0169 & 0.9998 \end{array}\right]$ ± $\left[\begin{array}{c} 4.3043\times 10^{-4}\\ 4.6892\times 10^{-4}\\ 2.1396\times 10^{-4} \end{array}\right]$	
*T*	$\left[\begin{array}{l} -0.0257\\ 0.0005\\ 0.0037 \end{array}\right]$		$\left[\begin{array}{l} 0.0262\\ 0.0001\\ -0.0002 \end{array}\right]$ ± $\left[\begin{array}{r} 0.00005\\ 0.00005\\ 0.00021] \end{array}\right]$		$\left[\begin{array}{l} 0.0265\\ -0.0007\\ -0.0030 \end{array}\right]$ ± $\left[\begin{array}{c} 1.7632\times 10^{-4}\\ 1.3328\times 10^{-4}\\ 2.0493\times 10^{-4} \end{array}\right]$	

**Table 5:** Calibration results for Microsoft Kinect V2.

	Burrus		Bouguet	
	RGB Camera	IR Camera	RGB Camera	IR Camera
$f_{x}$	1669.54	364.92	1057.58±1.83	369.15±0.66
$f_{y}$	1588.27	364.29	1055.33±1.77	368.01±0.64
$c_{x}$	345.85	256.67	971.26±1.71	260.68±0.53
$c_{y}$	251.59	205.33	538.00±1.68	205.92±0.60
$k_{1}$	−0.0343	0.0934	0.0413±0.0023	0.0631±0.0034
$k_{2}$	0.0697	−0.2748	−0.0389±0.0019	−0.1758±0.0044
$k_{3}$	−0.0257	0.0963	0	0
$p_{1}$	−0.0209	−0.0004	−0.00107±0.00044	−0.00096±0.00037
$p_{2}$	−0.0518	0.00004	0.00001±0.00049	−0.00062±0.00032
*R*	$\left[\begin{array}{lll} 0.9261 & -0.0471 & -0.3740\\ -0.0258 & 0.9818 & -0.1879\\ 0.3761 & 0.1837 & 0.9081 \end{array}\right]$		$\left[\begin{array}{l} 0.00156\\ 0.00402\\ -0.00691 \end{array}\right]$ ± $\left[\begin{array}{c} 0.00087\\ 0.00112\\ 0.00013 \end{array}\right]$	
*T*	$\left[\begin{array}{l} -0.0468\\ 0.0080\\ -0.3432 \end{array}\right]$		$\left[\begin{array}{l} 0.05211\\ -0.00061\\ -0.00319 \end{array}\right]$ ± $\left[\begin{array}{c} 0.00011\\ 0.00011\\ 0.00040 \end{array}\right]$	

## Appendix A

### Appendix A.1. Intrinsic Parameters

Intrinsic parameters refer to the internal camera geometric and optical characteristics. Focal length, principal point, and distortion are the most common parameters that represent the visual capability of a camera lens.

Usually, the focal length and the principal point are represented in a 3×3 matrix which is named the intrinsic matrix (Equation (A1)) where $f_{x}$, $f_{y}$, $c_{x}$ and $c_{y}$ are the focal length and the principal point, respectively.

(A1) $$\left[\begin{array}{ccc} f_{x} & 0 & c_{x}\\ 0 & f_{y} & c_{y}\\ 0 & 0 & 1 \end{array}\right]$$

For a given pixel *(u,v)* of the depth image with depth $Z_{d}$, the coordinates of a point *P* is obtained with the Equation (A2), being $f_{xd},f_{yd}$ the focal length and $c_{xd},c_{yd}$ the principal point of the infrared camera.

(A2) $$\begin{array}{c} P_{x}=\frac{\left(u-c_{xd}\right)Z_{d}}{f_{xd}}\\ P_{y}=\frac{\left(v-c_{yd}\right)Z_{d}}{f_{yd}}\\ P_{z}=Z_{d} \end{array}$$

The distortion is given by the first terms of the Taylor series, but it could be simplified to the Equation (A3) for the radial distortion and Equation (A4) for the tangential [69], where (i,j) is the distorted position of a pixel in the image, *r* is the radius to the principal point and $i_{c},j_{c}$ is the corrected position. For more information about distortions, see the work of Weng et al. [70].

(A3) $$\begin{array}{c} i_{c}=i\cdot \left(1+k_{1}r^{2}+k_{2}r^{4}+k_{3}r^{6}\right)\\ j_{c}=j\cdot \left(1+k_{1}r^{2}+k_{2}r^{4}+k_{3}r^{6}\right) \end{array}$$

(A4) $$\begin{array}{c} i_{c}=i+\left[2p_{1}y+p_{2}\left(r^{2}+2x^{2}\right)\right]\\ j_{c}=j+\left[p_{1}\left(r^{2}+2y^{2}\right)+2p_{2}x\right] \end{array}$$

### Appendix A.2. Extrinsic Parameters

Extrinsic parameters refer to the relative position and orientation of the camera frame with respect to a world coordinate system. Specifically, in a multiple camera system, multiple images are obtained using different coordinate systems. In this case, the extrinsic parameters describe the geometric relationship between the cameras that might be needed to align the multiple images into a common coordinate system. Usually, using the extrinsic parameters of each camera (rotation and translation) and the calculated baseline, and the orientation between cameras, it is possible to correlate the information from multiple sources.

The extrinsic parameters are a 3×3 rotation matrix and a translation vector of size 3×1. To align images from RGB-D devices, the point cloud is transformed and is projected on the colour sensor. Given a three dimensional point *P* of the point cloud, the transformations are applied through the Equation (A5).

(A5) $$P^{\prime }=R*P+T$$

where *R* and *T* are the rotation and translation matrices, respectively, and $P^{\prime }$ is the transformed 3D point of the point cloud. Next, the data is re-projected on the colour sensor using its intrinsic parameters (Equation (A6)).

(A6) $$\begin{array}{c} i=\left(\frac{P^{\prime }.x\cdot f_{xrgb}}{P^{\prime }.z}\right)+c_{xrgb}\\ j=\left(\frac{P^{\prime }.y\cdot f_{yrgb}}{P^{\prime }.z}\right)+c_{yrgb} \end{array}$$

where $f_{xrgb}$, $f_{yrgb}$, $c_{xrgb}$ and $c_{yrgb}$ are the focal length and principal point of the RGB camera. Applying these equations the bidimensional coordinates (i,j) in the colour image corresponding to that 3D point (*P*) of the point cloud are obtained.
